# UPLC-MS/MS Analysis of Hydroxyanthracene Derivatives in Botanical Food Products and Supplements: Surveillance of the Italian Market

**DOI:** 10.3390/foods14071229

**Published:** 2025-03-31

**Authors:** Mariantonietta Peloso, Alessandro Capriotti, Damiano Accurso, Elena Butovskaya, Giorgio Fedrizzi, Elisabetta Caprai

**Affiliations:** 1 National Reference Laboratory for Plant Toxins in Food, Food Chemistry Department, Istituto Zooprofilattico Sperimentale della Lombardia e dell’Emilia Romagna “Bruno Ubertini” (IZSLER), Via P. Fiorini 5, 40127 Bologna, Italy; m.peloso@izsler.it (M.P.); alessandro.capriotti@izsler.it (A.C.); damiano.accurso@izsler.it (D.A.); giorgio.fedrizzi@izsler.it (G.F.); 2 Food and Feed Chemistry Department, Istituto Zooprofilattico Sperimentale della Lombardia e dell’Emilia Romagna “Bruno Ubertini” (IZSLER), Via A. Bianchi 9, 25124 Brescia, Italy; elena.butovskaya@izsler.it

**Keywords:** hydroxyanthracene derivatives, LC-MS/MS, validation, *Aloe vera*, *Cassia*, *Rheum*, *Rhamnus*, food supplements, beverages, botanical food products

## Abstract

Hydroxyanthracene derivatives (HADs) are natural compounds that occur in several botanical species belonging to the genera *Aloe* L., *Cassia* L., *Rheum* L., *Frangula* Mill., and *Rhamnus* L. While they are widely used for their laxative effects, concerns persist about their potential genotoxicity and carcinogenicity. This study presents the development, validation, and application of a sensitive and rapid LC-MS/MS method to detect HAD (aloins, aloe-emodin, emodin, and danthron) levels in botanical food products and supplements. The method was validated according to criteria established by the European Union Reference Laboratory for Mycotoxins and Plant Toxins, and Regulation (EU) No 2783/2023 and was demonstrated to be fit-for-purpose. It was applied to 43 samples collected from the Italian market, including beverages, liquid and solid food supplements, herbal infusions, and jam based on aloe, senna, cassia, rhubarb, and frangula. The results revealed that 33% of the analyzed samples contained detectable HAD concentrations above the limit of quantification (LOQ = 0.5 mg/kg). The highest concentrations, up to 1352.9 mg/kg for the sum of aloin A and B, were found in solid food supplements derived from senna, cascara, rhubarb, and frangula. Aloe-emodin reached 213.4 mg/kg in a solid food supplement sample containing the same plant species, while the maximum detected concentration of emodin was 259.7 mg/kg in a senna-based supplement. No sample contained danthron. Conversely, in the majority of aloe beverage samples, HAD levels were found to be below the LOQ. In order to ensure the safety of consumers, it is essential that a more rigorous market surveillance of botanical food products is implemented, along with further toxicological studies.

## 1. Introduction

### 1.1. Hydroxyanthracene Derivatives: Botanical Sources, Pharmacological Properties, and Applications

Hydroxyanthracene derivatives (HADs) are a class of naturally occurring chemical compounds found in several plant species, such as *Aloe barbadensis* Miller, commonly known as *Aloe vera* L., *Senna alexandrina* Mill., *Rheum palmatum* L., *Rhamnus purshiana* D.C., and others described in Table 1 [1,2].

These organic compounds belong to the anthraquinone (AQ) family, derived from the anthracene structure with hydroxyl groups (-OH) attached, forming a characteristic triaromatic ring structure [1].

In nature, anthraquinones are found in various organisms, including fungi (e.g., *Penicillium* and *Aspergillus* species), lichens, and plants, and rarely in insects. In plants, AQs predominantly exist as glycosides, though they also occur in their free aglycone form. The aglycones identified in plants include emodin, rhein, chrysophanol, aloe-emodin, and physcion [3,4,5], as well as aloin, a mixture of diastereoisomers aloin A and B, present in aloe extracts [6].

Generally, hydroxyanthraquinones are present in plant extracts as pharmacologically inactive glycosides. However, certain compounds, such as aloin A or B (C-glycosides), undergo in vivo activation through glycosidic cleavage by the intestinal microbiota, leading to the formation of aloe-emodin [7]. Aloins are resistant to acid hydrolysis in the stomach and reach the large intestine intact, where bacterial enzymes metabolize them into glucose and aloe-emodin-9-anthrone, which is then oxidized to aloe-emodin [8].

These botanicals are frequently used in herbal infusions, food supplements, and other products, primarily for their laxative properties [9]. Their pharmacological activity is largely attributed to HADs’ ability to stimulate intestinal motility and reduce water and salts absorption in the intestine, thereby increasing stool volume [1].

Beyond their laxative effects, these plants exhibit a wide range of pharmacological activities due to the presence of various bioactive compounds, including polyphenols, flavonoids, and anthocyanins, which contribute to their antioxidant and anti-inflammatory properties [10].

In particular, *Aloe vera* is a plant with a long history of use in a wide variety of products due to its multiple health benefits. It is used in food, beverages, food supplements, cosmetics, and pharmaceuticals and has several recognized effects, such as anti-inflammatory, analgesic, immunomodulatory, laxative, antioxidant, anti-ulcerogenic, anti-irritant, antimicrobial, and anticancer [11].

HADs are predominantly concentrated in the latex portion of the *Aloe vera* leaf, contributing to the yellow, orange, or red coloration of the extract depending on their concentration [12]. To limit the presence of these compounds in commercial aloe products, two main processing methods are employed. The first isolates the inner gel of the parenchyma, while the second uses the whole leaf followed by filtration with activated charcoal [13,14].

Similarly, other HAD-containing plant species exhibit additional biological activities beyond their well-known applications. Frangula, for example, has demonstrated antimicrobial, antifungal, and insecticidal properties, which extend its use beyond medicinal applications to agricultural practices [15]. Likewise, cassia species are recognized for their antibacterial, antifungal, and antioxidant properties [16]. Rhubarb and senna exhibit numerous pharmacological effects, including hypoglycemic, hepatoprotective, and anti-inflammatory activities, primarily due to the presence of sennosides [17].

### 1.2. Safety Concerns and Regulatory Aspects

Hydroxyanthracene derivatives exert a range of biological effects, some of which have been associated with an elevated risk of severe adverse events [9].

In 2013, the European Food Safety Authority (EFSA) raised concerns regarding the prolonged use of HAD-containing laxatives, highlighting the potential risks of electrolyte imbalances, bowel dysfunction, and dependence [18]. In response to these concerns, EFSA was tasked by the European Commission to assess the safety of hydroxyanthracene derivatives following reports from EU Member States regarding their potential harmful effects, including a possible association with colorectal cancer (CRC).

In 2018, EFSA concluded that certain HADs, including emodin, aloe-emodin, and danthron (and *Aloe vera* extracts), are genotoxic in vitro, and aloe-emodin is genotoxic in vivo. Additionally, aloe leaf extracts and danthron are carcinogenic. EFSA also noted that human exposure to aloe-emodin and emodin is poorly characterized due to a lack of data on consumption patterns and concentrations in commercial products. A safe daily intake of HADs has not been established [1].

Concerns regarding the safety of HAD-containing products have also been raised by other regulatory agencies worldwide. The European Medicines Agency (EMA) confirmed that short-term use of HAD-containing laxatives is generally safe for occasional constipation but highlighted potential carcinogenic risks. They recommended against use in children under 12 and during pregnancy or lactation. Other organizations, including the German Federal Institute for Risk Assessment (BfR) and the International Agency for Research on Cancer (IARC), also raised concerns about the carcinogenic potential of anthranoids in *Aloe vera* products. Furthermore, the World Health Organization (WHO) recommended limiting the use of anthraquinone-containing products to one to two weeks and avoiding them in vulnerable groups [1].

In 2021, the European Commission adopted Regulation (EU) No 2021/468, amending Annex III of Regulation (EC) No 1925/2006 to add aloe-emodin, emodin, danthron, and aloe leaf extracts containing HADs to the list of prohibited substances in foods (Part A of Annex III). There is also growing concern about the health risks associated with preparations derived from *Rheum*, *Cassia,* and *Rhamnus*, though scientific uncertainty persists regarding the presence of HADs in these products [19].

Despite ongoing concerns, the scientific community remains divided on the genotoxicity of HADs. Some studies have shown no genotoxic effects in vivo of aloe-emodin and *Aloe ferox* resin on kidney and colon cells in mice [20,21]. Conversely, another study highlighted that aloe-emodin induced primary DNA damage in liver and kidney cells, suggesting an in vivo genotoxic mechanism [22]. Recently, other studies have suggested that whole plant extracts may exhibit reduced genotoxicity compared to isolated compounds. For example, rhubarb extract containing HADs showed no genotoxic activity in in vitro assays [23]. A research study using human colorectal adenocarcinoma cells (Caco-2) has further highlighted that whole plant extracts and individual molecules can have distinct effects on cell viability and protein expression, emphasizing the importance of considering the phytocomplex [24].

However, further research is needed to establish the safety of HADs in various botanical species [2]. Additionally, there is an urgent need for further toxicological studies and the development of reliable analytical methodologies to monitor these compounds in market products.

Meanwhile, the European Commission, based on the opinion of the European Union Reference Laboratory (EURL) for Mycotoxins and Plant Toxins, has established that the level of 1.0 mg/kg for aloe-emodin, emodin, and the sum of aloin A and aloin B is the lowest quantifiable level across EU laboratories. This quantification limit is considered appropriate for a harmonized risk management approach at the community level [25].

### 1.3. Analytical Approaches: State-of-the-Art and Objectives

Dietary exposure to HADs is primarily due to their use as laxatives in food supplements, which is why most quantification studies focus on these products [26,27]. Moreover, research has investigated their levels in dried extract of botanical species known to produce them [28,29,30,31,32,33], as well as in traditional Chinese medicine plants [34,35] and aloe-based beverages [36,37]. However, few studies have investigated their presence in herbal infusions [26], vegetables [38], and other plant-based products [39].

In the literature, only a limited number of validated methods have been applied to botanical food products and supplements for the determination of HADs and their glycoside derivatives. The most selective methods rely on LC-MS techniques, including LC-MS/MS [38,40], ultra-high-performance liquid chromatography-tandem mass spectrometry (UHPLC-MS/MS) [26,36], UPLC-MS/MS [41], and liquid chromatography with diode-array detection and mass spectrometry (LC-DAD-MS) [27]. Gas chromatography-mass spectrometry (GC-MS) [42] has also demonstrated high sensitivity, enabling the detection of very low levels of aloins in aloe-based products.

Considering the lack of analytical methods that address multiple types of products, the National Reference Laboratory for Plant Toxins in Food (NRL-TVN) at the Food Chemical Department of the Istituto Zooprofilattico Sperimentale della Lombardia e dell’Emilia-Romagna (IZSLER, Bologna) developed a research project to perform an UPLC-MS/MS method to monitor HADs (aloin A, aloin B, emodin, aloe-emodin, and danthron) in botanical food products and supplements. The method was validated and demonstrated to be fit-for-purpose, providing the advantage of rapid analysis while ensuring efficient extraction of all molecules under investigation with excellent recovery rates. It was applied to a total of 43 samples, including food supplements, herbal infusions, jam, and beverages derived from botanical species containing hydroxyanthracene derivatives.

## 2. Materials and Methods

### 2.1. Sampling

**Figure 1:**
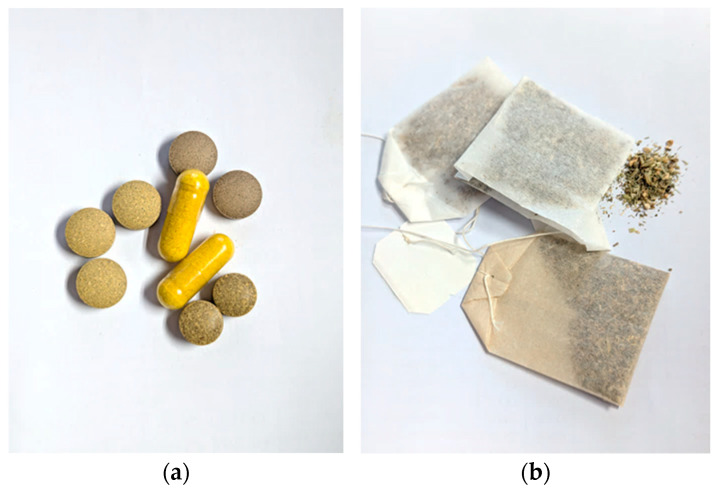
(**a**) Solid food supplement samples; (**b**) herbal infusion samples.

**Figure 2:**
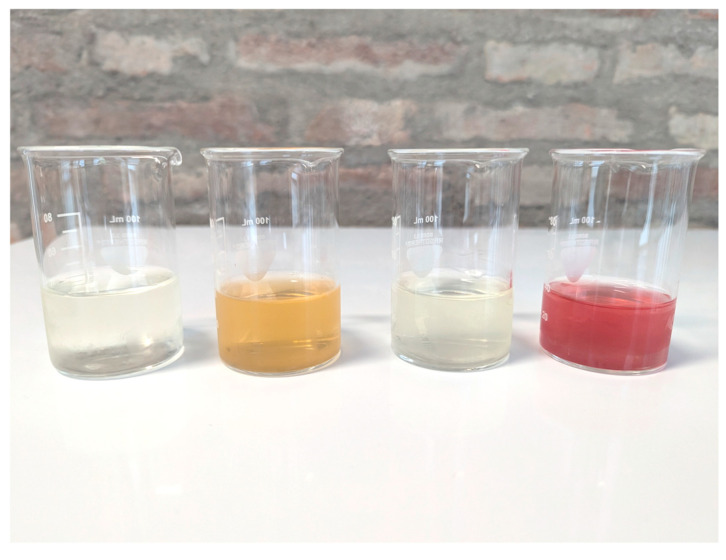
Aloe beverage samples.

The samples, comprising packaged food items, were collected from the Italian market. A total of 43 samples were analyzed, including various food categories such as beverages (n = 19), food supplements in both solid (n = 11) and liquid (n = 6) forms, herbal infusions (n = 6), and jam (n = 1). Images of some representative samples are shown in Figure 1 and Figure 2. The samples consisted mainly of the target botanical species in which hydroxyanthracene derivatives occur naturally, such as aloe, senna, cascara, frangula, and rhubarb.

Among these, beverage samples contained aloe at different concentrations, classified as “beverages” (1–30% aloe content, n = 7) and “aloe beverages” (>60% aloe content, n = 12). The only jam sample was aloe-based. The solid food supplements and herbal infusions comprised plant mixtures, most containing at least two target species, while some did not include any. Similarly, except for one sample containing aloe, all liquid food supplements were composed of non-target species. A detailed description of the samples is provided in Table S1.

### 2.2. Standards and Reagents

All reagents and solvents were of analytical grade. Reference standards of aloin A, aloin B, emodin, aloe-emodin, and danthron (Table 2) were purchased from PhytoLab GmbH & Co. KG (Vestenbergsreuth, Germany). Each standard showed a purity of ≥95%. Reference standard solutions were prepared at a concentration of 1 mg/mL in acetone, purchased from Carlo Erba Reagents (Val de Reuil, France). Solutions containing HADs mixture (10 µg/mL and 1 µg/mL) were prepared in methanol, purchased from VWR Chemicals (Rosny-sous-Bois, France). Subsequently, a calibration curve was prepared in the range 1.0 ng/mL–50.0 ng/mL. At each point on the calibration curve, 1,8-dihydroxyanthraquinone-D4 (danthron-D4), purchased from Toronto Research Chemicals (TRC, North York, Toronto, ON, Canada), was added as an internal standard (ISTD) at a concentration of 10 ng/mL. The ultrapure water (UPW) used for analysis was obtained from Evoqua Water Technologies (Pittsburgh, PA, USA). Formic acid 99% (HCOOH) and acetonitrile (ACN) of LC-MS grade, for the preparation of mobile phases, were acquired from Carlo Erba Reagents (Val de Reuil, France).

### 2.3. Sample and Quality Control Preparations

The liquid samples were subjected to manual shaking, whereas the solid samples were homogenized through the use of a blade mill or a mortar and pestle. The samples were then weighed (1 g ± 0.1 g) into 50 mL falcon-type tubes. An internal standard (Dantron-D4) at a concentration of 1 mg/kg was added to each sample. Subsequently, 10 mL of methanol was added to the sample, vortexed, and then sonicated for 15 min in an ultrasonic bath [27,44]. Thereafter, horizontal shaking was applied for further 15 min.

Subsequently, the samples were processed by centrifugation and dilution and analyzed without any further manipulations [24,40]. Specifically, the centrifugation was performed at 4000 rpm for 10 min at room temperature, and the supernatant was diluted in vials with methanol at varying proportions, depending on the product type: for beverages, the dilution factor was 500×, while for food supplements, it was 2000×. The vials were then vortexed and analyzed by LC-MS/MS.

For each matrix type subjected to analysis, quality control (QC) samples were prepared and processed in parallel with the test samples. These included a blank matrix and a fortified blank matrix with a solution of HADs at the limit of quantification (LOQ: 0.5 mg/kg).

### 2.4. Instruments and Analytical Conditions

The analyses were performed using a liquid chromatography system consisting of an UPLC Acquity I Class coupled with a Xevo TQ-Xs mass spectrometer (Waters) equipped with an UPLC Acquity BEH Phenyl 1.7 μm 2.1 mm × 100 mm (Waters) column, employing as mobile phases: water/acetonitrile 95:5 (*v*/*v*) with 0.1% formic acid (A) and acetonitrile with 0.1% formic acid (B). The flow rate was 0.35 mL/min with an injection volume of 5 µL. Gradient conditions were optimized to shorten the chromatographic run to 7 min. The binary gradient program was as follows: 0 min, 100% A; 0.5 min, 100% A; 5 min, 100% B; 6 min, 100% B; 7 min, 100% A.

The instrumental parameters were optimized through the continuous infusion of individual HADs in order to achieve the optimal instrumental conditions, as illustrated in Table 3. Subsequently, the two transitions with the highest intensity were selected (Table 4).

### 2.5. Validation Design

The LC-MS/MS method was validated for the detection of HADs (aloin A, aloin B, aloe-emodin, emodin, and danthron) and applied to food supplements, beverages, and botanical food products for concentrations of each analyte in the range from 0.5 to 10.0 mg/kg. The following parameters specified in the current legislation on sampling and analysis methods for the control of levels of plant toxins in food, in Regulation (EU) No 2783/2023 [45], and in the EURL-MP-guidance [46] were evaluated: linearity, limit of quantitation (LOQ), specificity/selectivity of the method, recovery, precision (repeatability, reproducibility), matrix effects (ME), and expanded measurement uncertainty (MU).

The linearity of the solutions used to prepare the calibration curve was assessed by evaluating the linear correlation coefficient (R^2^) and the deviation of the residuals by injecting at least three curves in the range 1 ng/mL–50 ng/mL with the internal extraction standard (Danthron-D4) at a concentration of 10 ng/mL.

The limit of detection (LOD) determination was based on the signal-to-noise (S/N) approach. The LOQ was established in line with the principles outlined by the European Commission and the EURL for mycotoxins and plant toxins, according to which the lowest concentration that can be reliably quantified in all EU laboratories is 1 mg/kg (EU-LOQ) for aloe-emodin and/or emodin and/or for the sum of aloin A and aloin B [25].

In order to evaluate the specificity of the method, 20 blanks for each matrix were analyzed to verify the absence of interference.

For beverages, 10 aloe-based beverages and 10 herbal infusion-based beverages from different brands and/or production batches were analyzed. For botanical food supplements, 20 different botanical food supplements from various brands and production batches were analyzed.

The % recovery was calculated for the matrices ‘beverages’ and ‘solid botanical food supplements’ using 54 fortified samples per matrix, with 6 replicates at three concentration levels over three days. Fortification levels were 0.5, 1.0, and 10.0 mg/kg for aloin A, aloin B, aloe-emodin, emodin, and danthron, and 1.0, 2.0, and 20.0 mg/kg for the sum of aloin A and B.

In terms of precision parameters, repeatability was assessed by analyzing six replicates of spiked blank samples at concentrations of 0.5, 1.0, and 10.0 mg/kg for individual analytes and 1.0, 2.0, and 20.0 mg/kg for the sum of aloin A and B in each sample. Within-laboratory reproducibility was evaluated using the same approach, with 18 samples spiked in six replicates per level, repeated on two additional occasions (days 2 and 3) under varying conditions. Matrix effects (ME) were assessed across 20 different product types by comparing the signal in the matrix for each hydroxyanthracene with that in the solvent for all prepared blanks at the same concentration.

The measurement uncertainty in the validation range of the method is expressed as the maximum relative expanded uncertainty (Û) using the bottom-up approach. This parameter was evaluated with a coverage factor of K = 2, and the effective degrees of freedom of the system were also assessed.

## 3. Results and Discussion

### 3.1. Method Development and Optimization

As a first sample preparation approach, a series of extraction tests were conducted using QuEChERS (Quick, Easy, Cheap, Effective, Rugged, Safe) salts to minimize the matrix effect, employing ethyl acetate and water as solvents. The extracts were subsequently evaporated under a gentle stream of nitrogen at 40 °C and diluted with methanol before being injected in UPLC-MS/MS. This procedure yielded high recoveries for most compounds, with the exception of aloin A and B, which likely resulted from their instability at elevated temperatures.

This phenomenon has been previously documented in the literature, where increased pH and temperature have been shown to induce the decomposition of aloin A [37,47]. Therefore, the extraction with QuEChERS was considered not suitable for further development. In order to avoid aloin degradation and optimize the development of a multi-analyte method—including aloin, aloe-emodin, emodin, and danthron—methanol was selected as the extraction solvent [27,44].

Analytical methods for the detection of HADs in botanical preparations typically involve extraction with methanol or ethanol, either alone or in aqueous solutions, followed by centrifugation (sometimes after sonication) and filtration before LC-MS/MS injection [23,24,26,27,37,40,42,48,49,50].

Following the approaches reported by Tinti et al. and Shi et al., the extracts were centrifuged and diluted up to 2000-fold [24,40].

The decision not to further manipulate the sample, such as by filtering it, was supported by the clarity of the highly diluted extract, which was free of visible particles. Indeed, key validation parameters support this choice, including the absence of peak overlapping in chromatograms, good repeatability, low relative standard deviation, satisfactory recovery, method linearity, and an evaluation of matrix effects.

To further improve accuracy in recovery assessment, an isotopically labeled internal standard, danthron-D4, was employed. This selection was driven by the unavailability of other labeled alternatives at the time; however, due to the structural and chemical similarities between danthron-D4 and the target analytes, excellent recoveries were achieved. Future work will focus on incorporating additional labeled compounds to expand the applicability of the method to a broader range of analytes. A limitation of the present study is its focus on a restricted number of molecules; future research will aim to include sennosides, glucofrangulins, physcion, and rhein.

To achieve optimal instrumental sensitivity, the mass spectrometer was operated in Multiple Reaction Monitoring (MRM) mode. Both positive (ESI+) and negative (ESI−) ionization modes were evaluated, as they are suitable for the HADs investigated. However, negative ion mode (ESI−) was selected as the precursor for most analytes due to reduced background interference, improving detection sensitivity. An exception was made for danthron, which was detected in positive mode (ESI+).

Overall, the methodological choices resulted in a simple and rapid extraction and quantification process, minimizing analyte degradation and enhancing result accuracy.

### 3.2. Performance Evaluation

The method was found to be fit for purpose, as all measured parameters were in in compliance with current regulatory requirements. The calibration curves were found to be linear, as indicated by a correlation coefficient of R^2^ = 0.999, a residue deviation of ≤20%, and the lowest value (1 ng/mL) of the curve being ≥3. The LOD and the LOQ for all studied matrices were 0.16 mg/kg and 0.5 mg/kg, respectively, for all individual compounds. The method is specific, as all interferents from the matrices were effectively removed, and no significant interfering peaks were observed in the chromatograms.

The mean % recovery for all analytes in beverages for each concentration level considered was 91.4%, and for the sum of aloin A and B, the mean recovery was 88.9%. For botanical food supplements, the mean recovery was 85.2%, and for the sum of aloin A and B, the mean recovery was 82.1%. The results were all found to be within the specified range of 70–120%, thereby demonstrating compliance with the stipulated requirements set out in Regulation (EU) No 2023/2783 [45]. Repeatability (RSD_r_) met regulatory criteria, remaining ≤20% across all matrices analyzed, and was expressed as a coefficient of variation (CV%). Within-laboratory reproducibility (RSD_WR_) also satisfied regulatory requirements, being ≤20%. The validation parameters were generated using the ADVeRSE 1.0 statistical application, a R package for processing the validation data of an analytical method [51]. The validation results for beverages and food supplements are presented in Tables S2 and S3.

### 3.3. Quantification

The identification and quantification of HADs was performed in accordance with SANTE/12089/2016 on the basis of the retention time of the analytes, the ion fragments, and the ion ratio. These were then compared to those from standard reference and control samples (blank samples fortified with HADs at the limit of quantification).

The presence of an analyte peak in the sample satisfies the following criteria: the retention time of each analyte in the sample is required to correspond to that of the solvent calibration curve (±0.1 min); the ionic ratio of the two monitored transitions for that analyte in the sample must be within ±30%; the signal-to-noise ratio (S/N) of the analyte peak in the sample must be at least ≥5. The matrix effect was assessed prior to validation procedures and evaluated as not relevant. All analytes had an instrumental response in a matrix-matched diluent that did not differ by more than 20% from that of a solvent. For this reason, HADs concentration was extrapolated by means of the least squares regression method based on a solvent calibration curve. LC-MS/MS chromatograms of five HADs are shown in Figure S1 [46,52].

### 3.4. Method Application: Results and Discussions

The validated method was applied to 43 samples for monitoring purposes, with results summarized in Table S4. Of these, 29 samples showed no detectable concentrations of HAD (<LOQ for each analyte; LOQ = 0.5 mg/kg). However, in the remaining 14 samples (33%), as shown in Table 5, varying concentrations of aloin A, aloin B, aloe-emodin, and emodin were detected. Specifically, the sum of aloin A and aloin B ranged from 1.0 mg/kg to 1352.9 mg/kg; aloe-emodin ranged from 0.8 mg/kg to 213.4 mg/kg (Figure S4); and emodin ranged from 1.0 mg/kg to 259.7 mg/kg (Figure S3). No sample contained danthron.

Among these 14 samples, the highest concentrations were found in solid food supplements. The composition of these food supplement samples included a variety of botanical species, such as cascara (*Rhamnus purshiana* D.C.) and frangula (*Rhamnus frangula* L.) bark, senna fruit (*Cassia angustifolia* M. Vahl), and rhubarb root (*Rheum palmatum* L.).

The average concentrations of the compounds were 449.1 mg/kg for the sum of aloin A and B, 33.5 mg/kg for aloe-emodin, and 63.2 mg/kg for emodin.

Regarding samples containing HADs, 10 included senna, 4 cascara, 4 rhubarb, and 2 frangula. Notably, only one aloe-based sample exhibited detectable levels of aloin A (0.5 mg/kg) and aloin B (0.5 mg/kg). As for herbal infusions, only one sample, based on senna and frangula, exhibited a high level of emodin (104.4 mg/kg) (Figure S5).

These results are in line with previous studies. For example, Loschi et al. analyzed the same set of molecules in botanical food supplements, beverages, and herbal infusions, detecting at least two molecules in the majority of samples at levels above 1 mg/kg [27]. Similarly, a study by Malysheva et al. found comparable concentrations of emodin (2.6–503.8 mg/kg) and aloe-emodin (12.41–149.46 mg/kg) in botanical food supplements, with some samples containing higher levels of other compounds, such as glucofrangulins, frangulins, and sennosides (up to 19.81 g/kg) [26].

Moreover, in the current study, two samples without target plant species showed HADs above the LOQ—an herbal infusion (emodin 2 mg/kg) and a solid food supplement (emodin 1 mg/kg). These products contained turmeric, *Curcuma longa* L. (Zingiberaceae family), another four plants belonging to the Asteraceae family (*Silybum marianum* L., *Cynara cardunculus var. scolymus* L., dandelion, and chamomile), and fennel (Apiaceae family). In relation to turmeric, several studies [53,54,55] on the phytochemical composition of the plant rhizomes identified anthraquinones among the many secondary metabolites. Moreover, there is a lack of research that has demonstrated the occurrence of emodin in turmeric such as in plants belonging to the Asteraceae family [38,56,57].

However, the specific origin of emodin in these samples remains uncertain; its presence may be a consequence of contamination with other botanical species during harvesting, as emodin is produced by fungi such as *Eurotium* and *Aspergillus* and is documented to occur frequently in agricultural crops [58].

In the beverage samples analyzed in this study, the HAD levels were below the LOQ, except for one sample (Figure S2), which had an aloins sum of 1.0 mg/kg. This findings aligns with results from other studies, such as the one by Di Minno et al., where the majority of the aloe beverages had aloin and aloe-emodin concentrations below 0.5 mg/kg [36].

Therefore, the absence or low concentrations of aloins and aloe-emodin in these samples suggest that these extracts were likely subjected to proper processing to minimize the HADs content.

The available literature focuses prevalently on the analysis of dried extracts from botanical species typically producing HADs [23,24,48] or on commercial aloe-based products [36,59,60]. Few studies, however, monitor commercial products—such as food supplements, beverages, infusions, and jams—derived from the target botanical species.

Obtaining new data on these products is essential for a more comprehensive assessment of consumer exposure to HADs and for ensuring rigorous monitoring to protect consumer safety. Additionally, further investigation is needed to clarify the occurrence of compounds such as emodin in specific products.

## 4. Conclusions

This study led to the development, validation, and accreditation of a reliable LC-MS/MS method for quantifying hydroxyanthracene derivatives in botanical food products and supplements. It met the validation criteria set by relevant regulations on plant toxins and the guidelines of the EURL Mycotoxin and Plant Toxins.

The method was applied to 43 samples collected from the Italian market, and the results revealed that 33% of the samples contained HAD concentrations exceeding the EU-LOQ of 1 mg/kg. Specifically, 7% were aloe-based beverages, 29% were senna-derived food supplements, 15% contained a blend of senna and cascara, and 14% were food supplements derived from a combination of all target plants.

Interestingly, two samples without any listed target botanical species contained HADs above 1 mg/kg, suggesting contamination during harvest or processing, or the presence of unidentified HAD sources. This emphasizes the need for further investigation into the potential occurrence of unlisted plant sources or contaminants.

These findings highlight the importance of ongoing monitoring, especially in products containing the targeted plants, and the need to explore other potential sources of HADs. Furthermore, the method will be extended to other molecules and other labeled compounds to enable the precise measurement of a wider range of analytes. To date, however, further toxicological studies are needed to better assess the health risks associated with the consumption of food products containing hydroxyanthracene derivatives.

## Figures and Tables

**Table 1:** HAD-containing botanical species.

Genus	Botanical Species	Common Names	Family	Anthraquinones	Parts Used
*Aloe* L.	*Aloe Vera* L. or *Aloe barbadensis* Miller, *Aloe ferox* Miller *Aloe africana* Mill.	Barbados aloe, cape aloe, African aloe	Aloaceae	Aloe-emodin, aloin A And aloin B	Leaf, leaf gel
*Rheum* L.	*Rheum palmatum* L., *Rheum officinale* Baillon	Chinese rhubarb	Polygonaceae	Emodin, palmidin C, rhein, sennoside A, sennoside B	Root and rhizome
*Cassia* L.	*Cassia fistula* L., *Cassia angustifolia* M. Vahl or *Senna alexandrina* Mill. or *Cassia senna* L.	Purging cassia, Alexandrian senna	Fabaceae/Leguminosae	Chrysophanol, physcion, rhein, sennoside B	Leaf and fruit
*Senna* Mill.	*Senna occidentalis* L. or *Cassia occidentalis* L.	Septicweed		Aloe-emodin, emodin, emodin anthrone and physcion	Bark, leaf and seed
*Frangula* Mill., *Rhamnus* L.	*Frangula alnus* Mill., or *Rhamnus frangula* L.	Frangula	Rhamnaceae	Emodin anthrone, glucofrangulin A, glucofrangulin B and palmidin C	Bark
	*Rhamnus purshiana* D.C.	Cascara buckthorn, sacred bark		Cascarosides, aloe-emodin and emodin	Bark

**Table 2:** Molecular formulae and chemical structures of hydroxyanthracene derivatives.

Analyte	Molecular Formula	Chemical Structure ^1^	CAS Number
Aloin A	C_21_H_22_O_9_	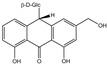	1415-73-2
Aloin B	C_21_H_22_O_9_	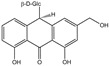	28371-16-6
Emodin	C_15_H_10_O_5_	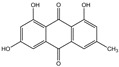	518-82-1
Aloe-emodin	C_15_H_10_O_5_	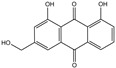	481-72-1
Danthron (1,8-Dihydroxyanthraquinone)	C_14_H_8_O_4_	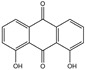	117-10-2
Danthron-D4 (1,8-Dihydroxyanthraquinone-D4)	C_14_H_4_D_4_O_4_	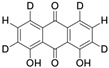	Not assigned

^1^ Drawing with ChemDraw 23.1.1 [43].

**Table 3:** Instrumental conditions.

MS/MS Parameters	Conditions
Ionization mode	ESI+/ESI−
Capillary voltage	2.0 kV (ESI+)/2.0 kV (ESI−)
Cone voltage	+40 V/−40 V
Source temperature	150 °C
Desolvation temperature	600 °C
Desolvation gas flow	(N_2_) = 1000 L/h
Collision gas flow	(Ar) = 0.17 mL/min

**Table 4:** LC-MS/MS parameters for all hydroxyanthracene derivatives.

HADs	Retention Time (min)	Precursor Ion (m/z)	Product Ion (m/z) Q/q ^1^	CE	Ionization Mode
Aloin B	2.80	417 [M−H]^−^	279/279	36/20	ESI (−)
Aloin A	2.86	417 [M−H]^−^	279/279	36/20	ESI (−)
Aloe-emodin	3.71	269 [M−H]^−^	211/240	20/20	ESI (−)
Emodin	4.10	269 [M−H]^−^	197/225	20/20	ESI (−)
Danthron	4.25	241 [M+H]^+^	121/139	25/35	ESI (+)
Danthron-D4	4.25	245 [M+H]^+^	143	40	ESI (+)

^1^ Q/q (Q = qualifier, q = quantifier).

**Table 5:** Samples with HAD concentrations above the LOQ, expressed in mg/kg.

Sample No	Sample Type	Aloin A	Aloin B	Sum: Aloin A + B	Aloe Emodin	Emodin	Danthron
**7**	Aloe beverage	0.5	0.5	1.0	<LOQ ^1^	<LOQ	<LOQ
**18**	Solid food supplement	29.5	29.9	59.4	<LOQ	19.3	<LOQ
**28**	Solid food supplement	742.3	385.9	1128.2	23.1	222.6	<LOQ
**29**	Solid food supplement	881.2	471.7	1352.9	27.5	259.7	<LOQ
**30**	Solid food supplement	28.2	23.2	51.4	0.9	8.2	<LOQ
**32**	Solid food supplement	<LOQ	<LOQ	<LOQ	213.4	43.1	<LOQ
**33**	Solid food supplement	<LOQ	<LOQ	<LOQ	0.8	36.4	<LOQ
**34**	Solid food supplement	<LOQ	<LOQ	<LOQ	<LOQ	1.0	<LOQ
**35**	Solid food supplement	<LOQ	<LOQ	<LOQ	3.0	<LOQ	<LOQ
**36**	Solid food supplement	66.4	35.4	101.8	5.9	51.4	<LOQ
**38**	Herbal infusion	<LOQ	<LOQ	<LOQ	23.5	104.4	<LOQ
**39**	Herbal infusion	<LOQ	<LOQ	<LOQ	<LOQ	2.0	<LOQ
**41**	Herbal infusion	<LOQ	<LOQ	<LOQ	12.5	4.1	<LOQ
**43**	Herbal infusion	<LOQ	<LOQ	<LOQ	24.2	6.3	<LOQ

^1^ LOQ = 0.5 mg/kg.

## Data Availability

The original contributions presented in the study are included in the article/Supplementary Material, further inquiries can be directed to the corresponding author.

## Supplementary Materials

The following supporting information can be downloaded at: www.mdpi.com/article/10.3390/foods14071229/s1, Figure S1: HADs chromatograms, 10 µg/L HADs mix + 10 µg/L ISTD; Figure S2. Chromatogram of aloe-emodin (213.4 mg/kg) in solid food supplement sample (Sample No 32); Figure S3. Chromatogram of emodin (259.7 mg/kg) in solid food supplement sample (Sample No 29); Figure S4. Chromatogram of emodin (104.4 mg/kg) in herbal infusion sample (Sample No 38); Figure S5. Chromatograms of aloin A (0.5 mg/kg) and aloin B (0.5 mg/kg) in aloe beverage sample (Sample No 7); Table S1: Sample list showing all ingredients and HAD source; Table S2: Validation parameters for each HAD at 3 spiking levels for beverages; Table S3: Validation parameters for each HAD at 3 spiking levels for botanical food supplements; Table S4: HAD concentrations found in all samples analyzed (mg/kg).
