# Supplementary material for: UPLC-MS/MS Analysis of Hydroxyanthracene Derivatives in Botanical Food Products and Supplements: Surveillance of the Italian Market
Source: Foods. 2025 Mar 31;14(7):1229. doi: 10.3390/foods14071229 (PMC11988599; doi:10.3390/foods14071229)
Supplement: Supplementary file 1 [file foods-14-01229-s001.zip › foods-3512999-supplementary.pdf]

foods

MDPI

Article

# UPLC-MS/MS Analysis of Hydroxyanthracene Derivatives in Botanical Food Products and Supplements: Surveillance of the Italian Market

**Mariantonietta Peloso [1], Alessandro Capriotti[1], Damiano Accurso[1], Elena Butovskaya[2], Giorgio Fedrizzi[1] and Elisabetta Caprai[1,*]**

[1] National Reference Laboratory for Plant Toxins in Food, Food Chemistry Department, Istituto Zooprofilattico Sperimentale della Lombardia e dell'Emilia Romagna "Bruno Ubertini" (IZSLER), Via P. Fiorini 5, 40127, Bologna, Italy.
[2] Food and Feed Chemistry Department, Istituto Zooprofilattico Sperimentale della Lombardia e dell'Emilia Romagna "Bruno Ubertini" (IZSLER), via A. Bianchi 9, 25124, Brescia, Italy.

* Correspondence: elisabetta.caprai@izsler.it

## Supplementary material

### 1. Figures

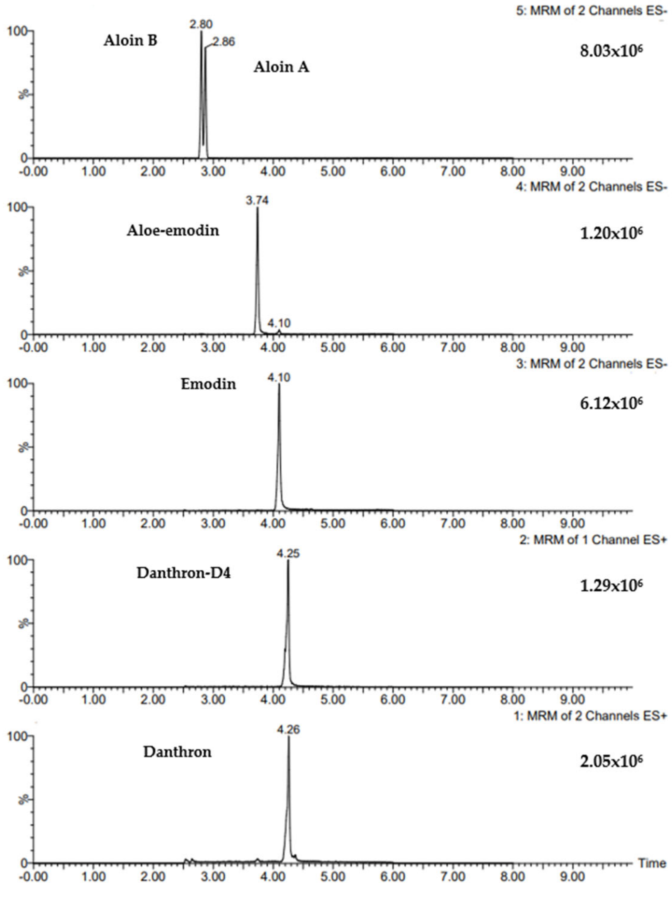


**Figure S1.** HADs chromatograms, 10 µg/L HADs mix + 10 µg/L ISTD

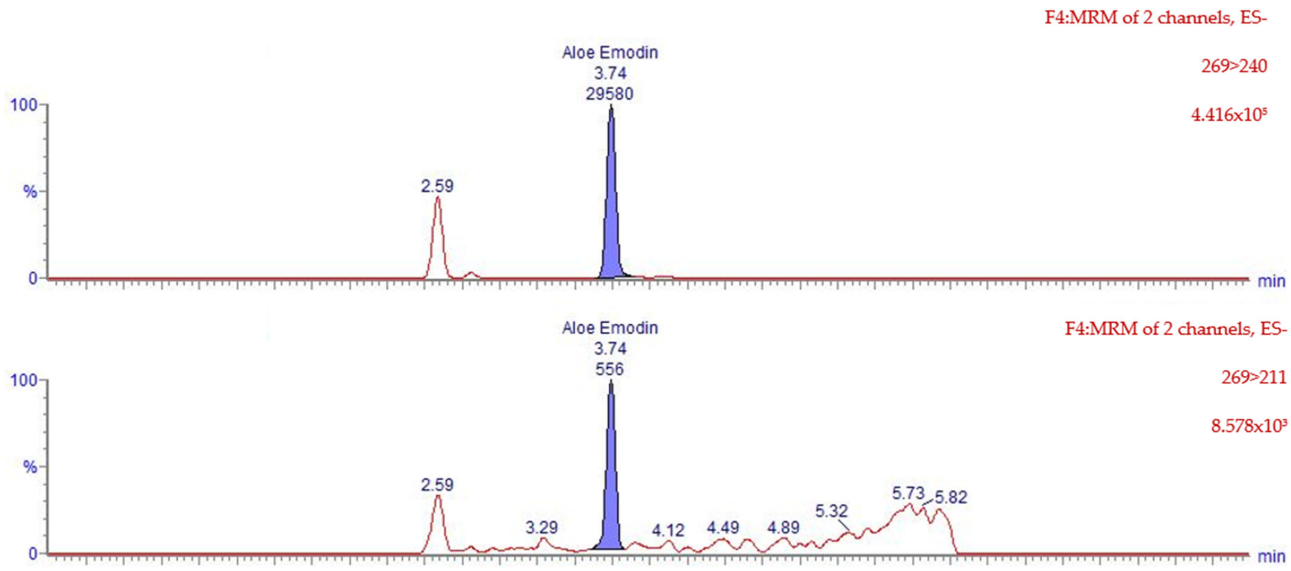


**Figure S2.** Chromatogram of aloe-emodin (213.4 mg/kg) in solid food supplement sample (Sample No 32)

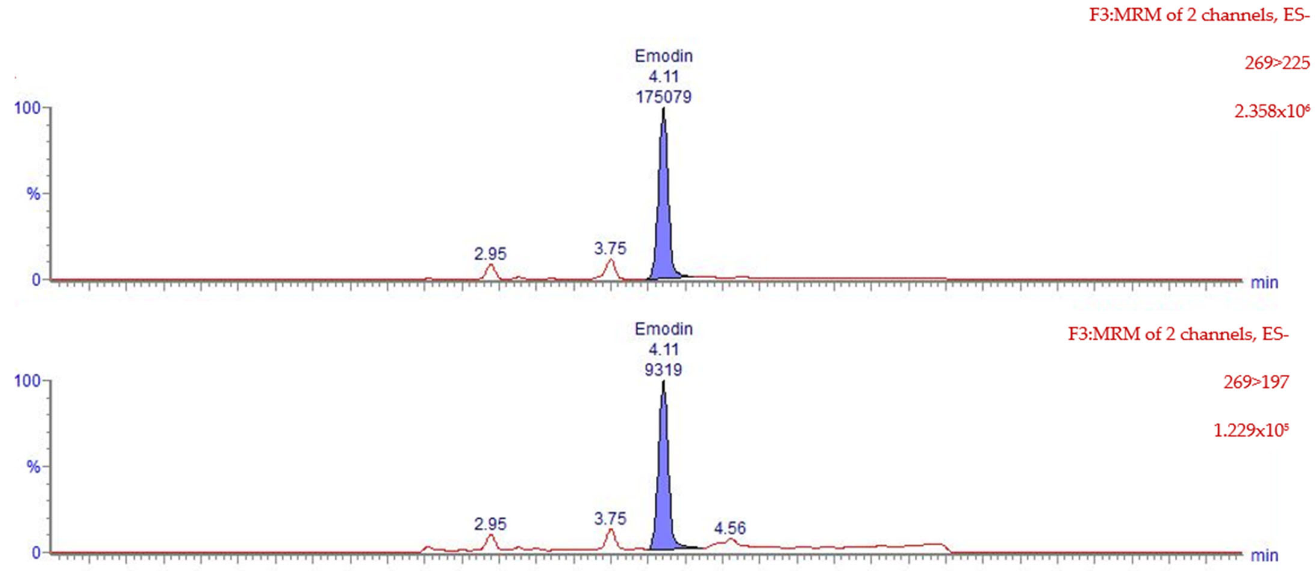


**Figure S3.** Chromatogram of emodin (259.7 mg/kg) in solid food supplement sample (Sample No 29**)**

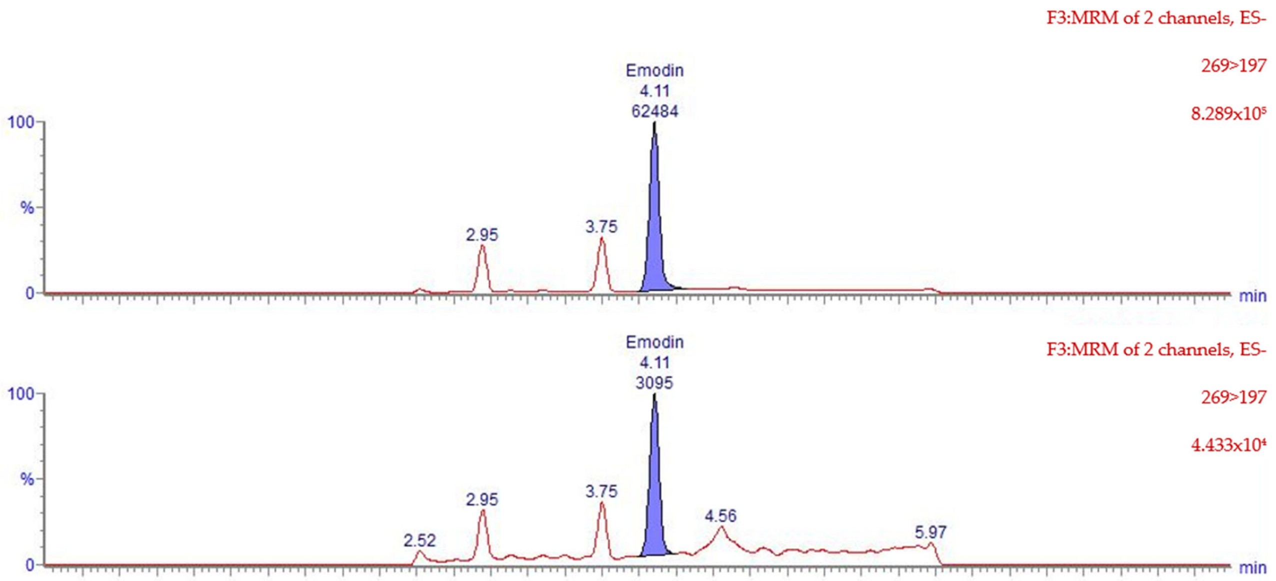


**Figure S4.** Chromatogram of emodin (104.4 mg/kg) in herbal infusion sample (Sample No 38)

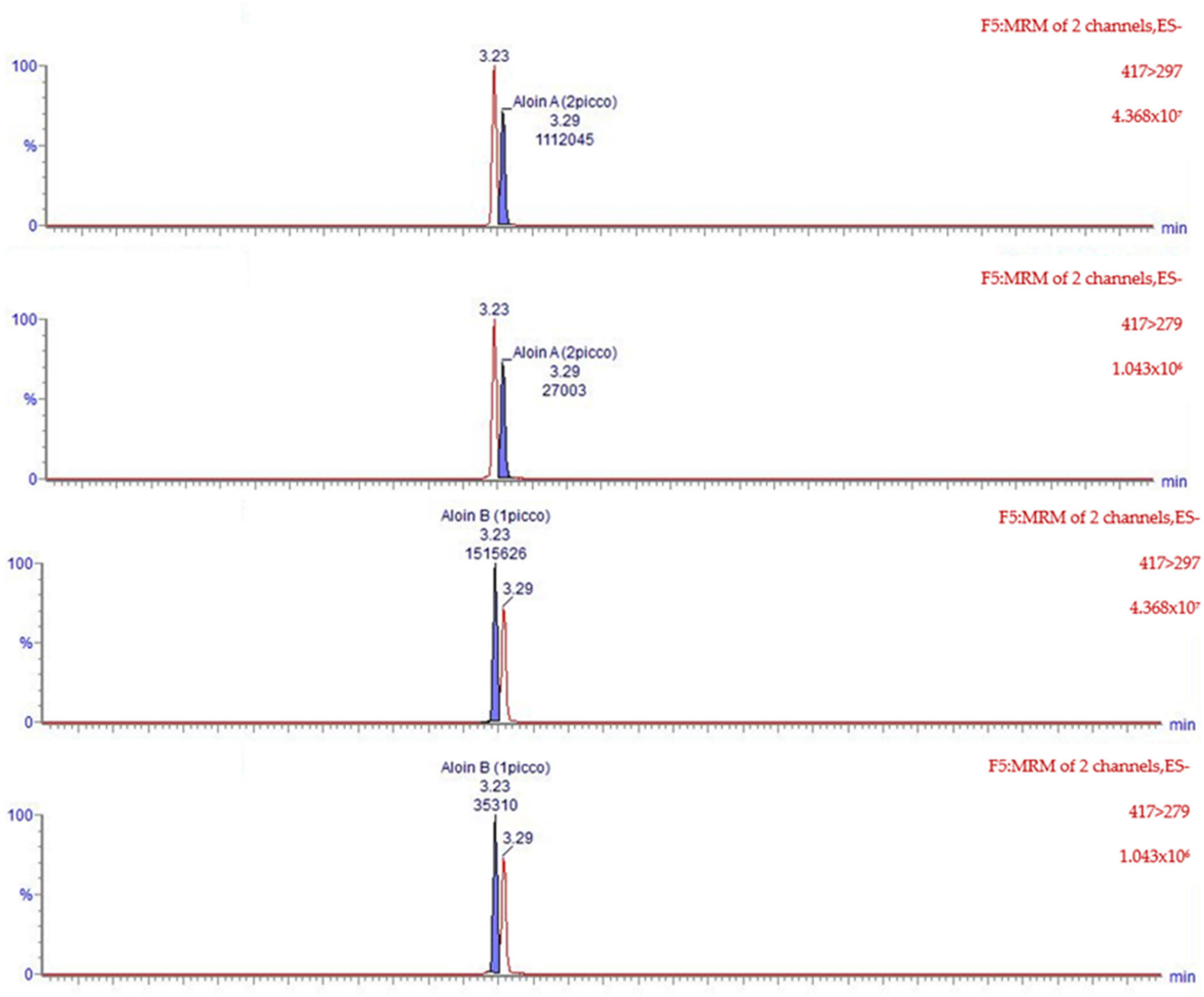


**Figure S5.** Chromatograms of aloin A (0.5 mg/kg) and aloin B (0.5 mg/kg) in aloe beverage sample (Sample No 7**)**

## 2. Tables

**Table S1.** Sample list showing all ingredients and HAD source

| Sample No | Sample Type | All Ingredients | HAD source |
|---|---|---|---|
| 1 | Beverage[1] | Water, 30% ***Aloe vera*** juice and pulp, cane sugar, acidity regulator: malic acid, grape aroma, stabilizers: calcium lactate; gellan gum, antioxidant: ascorbic acid. | Aloe Vera |
| 2 | Beverage | Purified water, **aloe** juice and pulp (*Aloe vera* L. Burm. - gelum sine cute, 30%), ginger juice (0.095%), fennel dry extract (*Foeniculum vulgare* Mill. - fruit) titrated at 1% in essential oil, lemon juice (0.005%), choline bitartrate, flavouring, antioxidant: L-ascorbic acid; acidifier: citric acid; acidity corrector: sodium citrate; | Aloe Vera |

| | | | |
|---|---|---|---|
| | | preservative: potassium sorbate; sweetener: sucralose; chromium picolinate. | |
| 3 | Aloe beverage[2] | **Aloe** juice and pulp (*Aloe vera* L. Burm. - gelum sine cute, 99.7%). Dry extract of Acerola (*Malpighia punicifolia* L. - fruit) titrated at 17% Vitamin C, acidity regulator: citric acid. | Aloe Vera |
| 4 | Beverage | Water, **aloe vera** juice and pulp (20%), acidity regulator: malic acid, grape aroma, stabilizers: calcium lactate; gellan gum, sweeteners: sucralose. | Aloe Vera |
| 5 | Aloe beverage | **Aloe Vera** (*Aloe barbadensis* Miller) standardized juice obtained from gel without cuticle; honey; aroma; Bilberry (*Vaccinium myrtillus* L.) fruit juice concentrate; acidifier: citric acid; preservatives: potassium sorbate and sodium benzoate. | Aloe Vera |
| 6 | Beverage | Water, 30% **aloe vera** juice and pulp, sugar, acidity regulator: malic acid, grape aroma, stabilizers: calcium lactate; gellan gum, antioxidant: ascorbic acid. | Aloe Vera |
| 7 | Aloe beverage | **Aloe Vera** (*Aloe barbadensis* Mill.) 100% gelum sine cute, 47.78g/99.55g, acidifier: citric acid; preservatives: sodium benzoate, potassium sorbate. | Aloe Vera |
| 8 | Beverage | Purified water, **aloe** juice and pulp (*Aloe vera* L.) Burm. - gelum sine cute, 30%. Black carrot juice from concentrate, 3.3%. Pomegranate juice powder, dried extract of pomegranate (*Punica granatum* L. - fruit) titrated at 20% in ellagic acid, choline bitrate, acidifier: citric acid; acidity corrector: sodium citrate; antioxidant: L-ascorbic acid; flavouring, preservative: potassium sorbate; sweetener: sucralose; sodium selenite. | Aloe Vera |
| 9 | Aloe beverage | **Aloe** (*Aloe vera* L. Burm. F., syn. *Aloe barbadensis* Mill.) gelum sine cute 60%, purified water, apple juice 4,85%, Birch (*Betula pendula* Roth., water, glycerine, citric acid) sap hydroglyceric extract, natural apple flavouring, preservatives: potassium sorbate, sodium benzoate; Birch (*Betula pendula* Roth., maltodextrin, silicon dioxide) leaves dry extract; acidifier: citric acid; sweetener: sucralose. | Aloe Vera |
| 10 | Aloe beverage | **Aloe** (*Aloe vera L*. Burm. F. syn. *Aloe barbadensis* Mill.) gelum sine cute 99%, acidulants: malic acid, citric acid, lactic acid. | Aloe Vera |
| 11 | Aloe beverage | **Aloe** (*Aloe vera* L. Burm. F. syn. *Aloe barbadensis* Mill.) gelum sine cute 60%, purified water, orange juice 4.85%, hydrolyzed collagen, sodium hyaluronate, natural flavouring, natural orange flavouring, calendula (*Calendula officinalis* L., maltodextrin, silicon dioxide) head dry extract tit. 1% flavonoids; preservatives: potassium sorbate, sodium benzoate; acidifier: citric acid; sweetener: sucralose. | Aloe Vera |
| 12 | Aloe beverage | **Aloe Vera** (*Aloe barbadensis* Miller) standardized juice obtained from gel without cuticle 100%; acidifier: citric acid; preservatives: potassium sorbate and sodium benzoate. | Aloe Vera |
| 13 | Beverage | Green tea (94%), natural lemon aroma (5%), dried and concentrated ***Aloe vera*** powder, 1%. | Aloe Vera |
| 14 | Aloe beverage | **Aloe** (*Aloe vera* L. Burm. F. syn. *Aloe barbadensis* Mill.) gelum sine cute, 99.55%; acidifier: citric acid; preservatives: sodium benzoate, potassium sorbate. | Aloe Vera |

| | | | |
|---|---|---|---|
| 15 | Jam | Pomegranate 70%, apple juice concentrate, **aloe** gel (*Aloe vera* L.) * 10%, hibiscus flowers(infusion) 1%, gelling agent: pectin. *bio, gluten free | Aloe Vera |
| 16 | Aloe beverage | **Aloe** (*Aloe vera* (L.) Burm. F. syn. *Aloe barbadensis* Mill.) gelum sine cute, (99.5%); acidifier: citric acid; preservatives: sodium benzoate, potassium sorbate. | Aloe Vera |
| 17 | Beverage | Water, 20% ***Aloe vera*** juice and pulp, acidity regulator: malic acid, grape aroma, stabilizers: calcium lactate, gellan gum, sweetener: sucralose. | Aloe Vera |
| 18 | Solid food supplement | Bulking agent: microcrystalline cellulose; anti-caking agents: magnesium stearate, silicon dioxide, tricalcium phosphate; fennel powder (*Foeniculum vulgare* Mill. - fruit), turmeric (*Curcuma longa* L.-rhizome), **senna** dry extract (*Cassia angustifolia* Vahl. -leaf) titrated at 20% sennoside B, bolbo powder (*Peumus bolbus Molina* -leaf), mallow powder (*Malva sylvestris* L. -leaf), camomile powder (*Matricaria chamomilla* L. -flower), liquorice dry extract (*Glycyrrhiza glabra* L. -root) titrated at 19% glycyrrhizic acid, vervain powder (*Lippia citriodora* Kunth -leaf), altea powder (*Althaea officinalis* L. -root), cumin powder (*Cuminum cyminum* L. -fruit), caraway powder (*Carum carvi* L. -fruit). Stabilizer: polyvinylpyrrolidone K; plum dry extract (*Prunus domestica* L. -fruit), hibiscus dry extract (*Hibiscus sabdariffa* L. -flower). Extracts contain maltodextrins. | Senna |
| 19 | Liquid food supplement | Apple juice concentrate, cane sugar, water, orange juice concentrate, artichoke (*Cynara scolymus*) leaves dry extract, rosemary (*Rosmarinus officinalis*) leaves dry extract, acacia gum, maté (*Ilex paraguariensis*) leaves dry extract, hawthorn (*Crataegus monogyna*) flowering tops dry extract, natural orange flavour, natural lemon flavour, thickener: xanthan gum. | No target ingredients |
| 20 | Liquid food supplement | Water, stabilizer: sorbitol syrup; watermelon (*Citrullus lanatus* (Thunb.) Matsum & Nakai) fruit dry extract, orange (*Citrus aurantium* var. dulcis Hayne) fruit juice concentrate, flavouring, kale (*Brassica oleracea* L.) leaves and flowers dry extract, hibiscus (*Hibiscus sabdariffa* L.) flowers dry extract, thickeners: acacia gum, xanthan gum; choline bitartrate, acidifier: citric acid; lemon (*Citrus limon* (L.) Osbeck) fruit juice concentrate, burdock (*Arctium lappa* L.) root dry extract tit. 2% inulin, preservatives: sodium benzoate, potassium sorbate; sweetener: sucralose. | No target ingredients |
| 21 | Liquid food supplement | Apple juice concentrate, **aloe** (*Aloe vera* L. Burm. F.) gelum sine cute juice, plum juice concentrate, inulin from chicory, gentiana (*Gentiana lutea* L.) root d.e. tit. 5-6% in caffeoylquinic acids expressed as chlorogenic acid, milk thistle phytosome (*Silybum marianum* (L.) Gaertn., soya lecithin) fruit dry extract tit. 0.04% protopine, birch (*Betula pendula* Roth) leaves dry extract tit. 2.5% flavonoids, stabilizer: acacia gum; preservative: potassium sorbate; thickener: xanthan gum; anise (*Pimpinella anisum* L.) fruit dry extract 4:1. | Aloe Vera |
| 22 | Liquid food supplement | Water; concentrated fig juice (*Ficus carica* L.), fruit; concentrated apple juice (*Malus domestica* Borkh.), fruit; fructo-oligosaccharides; milk thistle extract (*Silybum marianum* (L.) Gaertn.), seed husk; artichoke extract (*Cynara scolymus* L.), capitula; tamarind extract | No target ingredients |

| | | | |
|---|---|---|---|
| | | (*Tamarindus indica* L.), fruit pulp; preservatives: sodium benzoate, potassium sorbate. | |
| 23 | Liquid food supplement | Aqueous plant extract (obtained from 500 mg green tea leaves (*Camellia sinesis* L. Kuntzel), 450 mg ash leaves (*Fraxinus excelsiorius* L.), 450 mg fennel fruits (*Foeniculum vulgare* Mill.), 300 mg of cinnamon bark (*Cinnamomum zeylanicum* Blume), 250 mg of lemon balm rhizome (*Elymus repens* Gould.), 250 mg of black elder flowers (*Sambucus nigra* L.)], concentrated apple juice. | No target ingredients |
| 24 | Aloe beverage | Water, **aloe** (*Aloe vera* L. Brum. F.) gelum sine cute, hydroglyceric plant extracts (water, glycerol, plant part in ratio D:E 1:2) of: artichoke (*Cynara scolymus* L.) leaves, milk thistle (*Silybum marianum* L. Gaertn.) fruit, dandelion (*Taraxacum officinale* L. Weber ex F.H.Wigg.) root, green anise (*Pimpinella anisum* L.) fruit, burdock (*Arctium Iappa* L. ) root, Verbena (*Verbena officinalis* L.) leaves, juniper (*Juniperus communis* L.) galbules, birch (*Betula pendula* Roth) leaves, acidifier: ascorbic acid, preservative: potassium sorbate. | Aloe Vera |
| 25 | Aloe beverage | **Aloe** (*Aloe vera* L. Burm. F. syn. *Aloe barbadensis* Mill.) gelum sine cute 60%, purified water, apple juice 4,85%, birch (*Betula pendula* Roth., water, glycerine, citric acid) sap hydroglyceric extract, natural apple flavouring, preservatives: potassium sorbate, sodium benzoate; birch (*Betula pendula* Roth., maltodextrin, silicon dioxide) leaves dry extract; acidifier: citric acid; sweetener: sucralose. | Aloe Vera |
| 26 | Aloe beverage | **Aloe** (*Aloe vera* L. Burm. F. syn. *Aloe barbadensis* Mill.) gelum sine cute 60%, purified water, pineapple juice powder 4,85%, moringa (*Moringa oleifera* Lam.) seeds dry extract tit. 20% in glycosides; natural pineapple flavouring; preservatives: potassium sorbate, sodium benzoate; acidifier: citric acid; sweetener: sucralose. | Aloe Vera |
| 27 | Liquid food supplement | Water, Fluid extracts of: centella (*Centella asiatica* L. Urb.- herb) Artichoke (*Cynara scolymus* L. - leaf), milk thistle (*Silybum marianum* L. Gaertn. - fruit), turmeric (*Curcuma longa* L. - rhizome), fumaria (*Fumaria officinalis* L. - aerial part with flowers), juniper (*Juniperus communis* L. - seed), blackcurrant (*Ribes nigrum* L. - leaf), rosemary (*Rosmarinus officinalis* L. - leaf), green tea (*Camelia sinesis* L. Kuntze - leaf), fennel (*Foeniculum vulgare* Mill. - fruit); aroma, acidity corrector: citric acid, preservatives: potassium sorbate and sodium benzoate; Sweetener: sucralose. | No target ingredients |
| 28 | Solid food supplement | Mixture of powdered herbs: **cascara** (*Rhamnus purshiana* DC.) bark, **frangula** (*Rhamnus frangula* L.) bark, **senna** (*Cassia angustifolia* M. Vahl.) fruit, mallow (*Malva sylvestris* L. ) leaf, fennel (*Foeniculum vulgare* Miller) fruit, **rhubarb** (*Rheum palmatum* L.) root/rhizome, mint (*Mentha x piperita* L.) leaf, dandelion (*Taraxacum officinale* L. Weber ex F.H. Wigg.) root, cinnamon (*Cinnamomum verum* J. Presl. ) bark, lemon balm (*Melissa officinalis* L.) leaf; maltodextrins from maize; stabilizer: acacia gum; **senna** (*Cassia angustofolia* M. Vahl.) leaf dry extract tit. 7% hydroxyanthracene glycosides as sennoside B; anti-caking agents: magnesium salts of fatty acids and silicon dioxide; glazing agents: hydroxypropyl methyl cellulose, fatty acids, microcrystalline cellulose. | Cascara, Frangula, Senna, Rhubarb |

| | | | |
|---|---|---|---|
| 29 | Solid food supplement | Mixture of powdered herbs: **cascara** (*Rhamnus purshiana* DC.) bark, **frangula** (*Rhamnus frangula* L.) bark, **senna** (*Cassia angustifolia* M. Vahl.) fruit, mallow (*Malva sylvestris* L. ) leaf, fennel (*Foeniculum vulgare* Miller) fruit, **rhubarb** (*Rheum palmatum* L.) root/rhizome, mint (*Mentha x piperita* L.) leaf, dandelion (*Taraxacum officinale* L. Weber ex F.H. Wigg.) root, cinnamon (*Cinnamomum verum* J. Presl. ) bark, lemon balm (*Melissa officinalis* L.) leaf; maltodextrins from maize; bulking agent: microcrystalline cellulose; stabilizer: acacia gum; **senna** (*Cassia angustofolia* M. Vahl.) leaf d.e. tit. 7% hydroxyanthracene glycosides as sennoside B; anti-caking agents: magnesium salts of fatty acids and silicon dioxide; glazing agents: hydroxypropyl methyl cellulose, fatty acids, microcrystalline cellulose. | Cascara, Frangula, Senna, Rhubarb |
| 30 | Solid food supplement | Bulking agent: microcrystalline cellulose; anti-caking agents: magnesium stearate, silicon dioxide, tricalcium phosphate; fennel powder (*Foeniculum vulgare* Mill. - fruit), turmeric (*Curcuma longa* L.-rhizome), **senna** d.e. (*Cassia angustifolia* Vahl. -leaf) tit. 20% sennoside B, bolbo powder (*Peumus bolbus* Molina -leaf), mallow powder (*Malva sylvestris* L.-leaf), camomile powder (*Matricaria chamomilla* L. -flower), liquorice dry extract (*Glycyrrhiza glabra* L. -root) titrated at 19% glycyrrhizic acid, verbena powder (*Lippia citriodora* Kunth -leaf), altea powder (*Althaea officinalis* L. -root), cumin powder (*Cuminum cyminum* L. -fruit), **cascara** sagrada powder (*Rhamnus purshiana* DC. -bark), caraway powder (*Carum carvi* L. -fruit). stabilizer: polyvinylpyrrolidone K; plum dry extract (*Prunus domestica* L. -fruit), hibiscus dry extract (*Hibiscus sabdariffa* L. -flower), coriander dry extract (*Coriandrum sativum* L. -fruit). Extracts contain maltodextrins. | Senna, Cascara |
| 31 | Solid food supplement | Maltodextrin, cranberry dry extract (*Vaccinium macrocarpon* Aiton - fruit) tit. 1% proanthocyanidins, echinacea dry extract (*Echinacea purpurea* Moench - herb) tit. 4% polyphenols, vitamin C (ascorbic acid), anti-caking agents: magnesium stearate and silicon dioxide. | No target ingredients |
| 32 | Solid food supplement | **Senna**, chicory, fennel, dandelion, caraway and caraway. Gluten-free. | Senna |
| 33 | Solid food supplement | Green anise powder (*Pimpinella anisum* L. - fruit), fennel powder (*Foeniculum vulgare* Mill. - fruit), caraway powder (Carum carvi L. - fruit), **rhubarb** d.e. (*Rheum officinale* Baill. and *Rheum palmatum* L. - root) tit. 5% reine, stabiliser: microcrystalline cellulose, anti-caking agent: magnesium stereate. Vegetable capsule: hydroxypropyl methylcellulose. Contains maltodextrin. | Rhubarb |
| 34 | Solid food supplement | Turmeric powder (*Curcuma longa* L. - rhizome), milk thistle dry extract (*Silybum marianum* (L.) Gaertn. - fruit) tit. 2% in silymarin, stabilizer: microcrystalline cellulose, artichoke d.e. (*Cynara scolymus* L. - leaf) tit. 2.5% in caffeilquinic acids, anti-caking agents: magnesium stereate and silicon dioxide. Vegetable capsule: hydroxypropyl methylcellulose. Contains maltodextrin. | No target ingredients |
| 35 | Solid food supplement | **Senna** e.s. tit. 20% sennosides (*Cassia angustofolia* M. Vahl., fruit), bulking agents: dicalcium phosphate, microcrystalline cellulose; anti-caking agents: talc, cross-linked sodium carboxymethyl cellulose, silicon dioxide (nano), magnesium salts of fatty acids; | Senna |

| | | | |
|---|---|---|---|
| | | glazing agents: hydroxypropyl methylcellulose, shellac, microcrystalline cellulose, fatty acids. | |
| 36 | Solid food supplement | Bulking agent: microcrystalline cellulose, mallow powder (*Malva sylvestris* L-leaf), **senna** e.s. (*Cassia angustifolia* VAhl. - leaf) tit. 20% sennoside B, anti-caking agents: magnesium stereate and silicon dioxide; **Cascara** e.s. (*Rhamnus frangula* L. - bark) tit. 20% glucofrangulin. Extracts contain maltodextrin. | Senna, Cascara |
| 37 | Solid food supplement | Cane sugar*, gum arabic, natural mint flavouring. *Organic farming. | No target ingredients |
| 38 | Herbal infusion | **Frangula** (*Frangula alnus* Mill.) bark*, liquorice (*Glycyrrhiza glabra* L.) roots*, **senna** (*Senna alexandrina* Mill.) leaves*, mallow (*Malva sylvestris* L.) leaves and flowers*, fennel (*Foeniculum vulgare* Mill.) fruits*, stevia (*Stevia rebaudiana* Bertoni) leaves*. *bio, organic | Frangula, Senna |
| 39 | Herbal infusion | Dandelion* (32%), chamomile*, turmeric* (23%), fennel*. *Organic farming. | No target ingredients |
| 40 | Herbal infusion | Dandelion root (*Taraxacum officinale* L. Weber ex F.H. Wigg.)*, fennel fruit (*Foeniculum vulgare* Mill.)*, sage leaves (*salvia officinalis* L.)*, horsetail aerial parts (*Equisetum arvense* L.)*, nettle leaves (*Urtica dioica* L.)*, juniper berries (*Juniperus communis* L.)*. *Organic farming. | No target ingredients |
| 41 | Herbal infusion | Fennel fruit (*Foeniculum vulgare* Mill.) 30.6%, chamomile flowers (*Matricaria chamomilla* L.), **senna** leaves (*Cassia senna* L.) 12%, liquorice root (*Glycyrrhiza glabra* L.), orange leaves (*Citrus aurantium* subsp. *Sinesis* L., Engl.), flavourings, caraway fruit (*Carum carvi* L.) 5%, dietary fibre: inulin, green aniseed fruit (*Pimpinella anisum* L.) 3%, **rhubarb** root (*Rheum officinale* Baill.) 1%, prune juice granules. | Senna, Rhubarb |
| 42 | Herbal infusion | Fennel fruit (*Foeniculum vulgare* Mill.) 53%, lemongrass aerial part (*Cymbopogon schoenanthus* L., Spreng.) 21%, cinnamon bark (*Cinnamomum* ***cassia***, Ness &; T.Nees, J. Presl) 8%, caraway fruit (*Carum carvi* L.) 6%, green anise fruit (*Pimpinella anisum* L.) 5%, chamomile flowers (*Matricaria chamomilla* L.), ginger rhizome extract (*Zingiber officinale* Rosc.) 2%. | Cassia |
| 43 | Herbal infusion | **Senna** leaves 40% (Cassia Angustifolia Vahl.), inulin from chicory root 24% (*Cichorium Intybus* L.), dandelion leaves 20% (*Taraxacum Officinale* Weber), chamomile flowers (*Matricaria Chamomilla* L.), mint leaves (*Mentha x Piperita* L.), fennel fruits (*Foeniculum Vulgare* Mill.). | Senna |

[1]Beverage: aloe content is between 10-30%; [2]Aloe Beverage: aloe content is above 60%.

**Table S2**: Validation parameters for each HAD at 3 spiking levels for beverages

| Analyte | Spiking Level mg/kg | Mean Conc. mg/kg | S[1] | RSDr[2]-CV% | $RSD_{WR}$[3]-CV% | Mean Recovery % *Beverages* |
|---|---|---|---|---|---|---|
| **Aloin A** | 0.5 | 0.4 | 0.01 | 3.0 | 9.6 | 90.8 |
| | 1 | 0.9 | 0.02 | 3.0 | 10.2 | |

| | | | | | | |
|---|---|---|---|---|---|---|
| | 10 | 8.8 | 0.21 | 5.2 | 14.2 | |
| **Aloin B** | 0.5 | 0.4 | 0.02 | 4.2 | 7.2 | 87.0 |
| | 1 | 0.9 | 0.03 | 3.4 | 6.0 | |
| | 10 | 8.7 | 0.35 | 4.7 | 4.7 | |
| **Emodin** | 0.5 | 0.5 | 0.02 | 4.7 | 5.8 | 98.8 |
| | 1 | 1.0 | 0.02 | 2.1 | 3.8 | |
| | 10 | 10.9 | 0.59 | 6.2 | 7.2 | |
| **Aloe-emodin** | 0.5 | 0.4 | 0.02 | 4.4 | 5.5 | 91.3 |
| | 1 | 0.9 | 0.01 | 2.3 | 5.7 | |
| | 10 | 9.3 | 0.40 | 4.9 | 8.7 | |
| **Danthron** | 0.5 | 0.4 | 0.02 | 2.8 | 2.7 | 89.1 |
| | 1 | 0.9 | 0.02 | 1.5 | 2.1 | |
| | 10 | 9.3 | 0.24 | 3.6 | 3.4 | |
| **Aloin A+Aloin B** | 1 | 0.8 | 0.02 | 3.2 | 4.6 | 88.9 |
| | 2 | 1.7 | 0.05 | 2.9 | 4.3 | |
| | 20 | 17.5 | 0.53 | 4.8 | 8.6 | |

[1](S) Standard Deviation; [2] The Coefficient of Variation (CV%) of the repeatability; and [3]The Coefficient of Variation (CV%) of within-laboratory reproducibility were calculated by analyzing blank samples in six replicates at the three fortified levels.

**Table S3**: Validation parameters for each HAD at 3 spiking levels for botanical food supplements

| Analyte | Spiking Level mg/kg | Mean Conc. mg/kg | S[1] | RSDr[2]-CV% | $RSD_{WR}$[3]-CV% | Mean Recovery % *Food Supplements* |
|---|---|---|---|---|---|---|
| **Aloin A** | 0.5 | 0.4 | 0.06 | 11.2 | 10.4 | 89.0 |
| | 1 | 0.9 | 0.08 | 9.1 | 8.4 | |
| | 10 | 9.3 | 0.77 | 6.8 | 6.9 | |
| **Aloin B** | 0.5 | 0.3 | 0.1 | 20.7 | 19.1 | 75.5 |
| | 1 | 0.7 | 0.15 | 16.4 | 15.0 | |
| | 10 | 9.0 | 0.49 | 5.8 | 6.0 | |
| **Emodin** | 0.5 | 0.5 | 0.05 | 10.2 | 15.6 | 105.1 |
| | 1 | 1.3 | 0.09 | 7.5 | 11.2 | |
| | 10 | 11.7 | 0.61 | 6.9 | 12.7 | |
| **Aloe-emodin** | 0.5 | 0.4 | 0.04 | 7.6 | 7.8 | 83.0 |

| | | | | | | |
|---|---|---|---|---|---|---|
| | 1 | 0.9 | 0.08 | 7.5 | 7.0 | |
| | 10 | 8.6 | 0.06 | 4.6 | 5.8 | |
| **Danthron** | 0.5 | 0.3 | 0.03 | 4.2 | 7.1 | 73.4 |
| | 1 | 0.8 | 0.04 | 4.1 | 4.7 | |
| | 10 | 7.5 | 0.48 | 4.4 | 4.2 | |
| **Aloin A+Aloin B** | 1 | 0.7 | 0.1 | 11.4 | 10.5 | 82.1 |
| | 2 | 1.6 | 0.1 | 6.1 | 5.8 | |
| | 20 | 18.4 | 1.21 | 6.1 | 6.2 | |

[1](S) Standard Deviation; [2] The Coefficient of Variation (CV%) of the repeatability; and [3]The Coefficient of Variation (CV%) of within-laboratory reproducibility were calculated by analyzing blank samples in six replicates at the three fortified levels

**Table S4:** HAD concentrations found in all samples analyzed (mg/kg)

| Sample No | Sample Type | Aloin A | Aloin B | Sum: Aloin A+B | Aloe Emodin | Emodin | Danthron |
|---|---|---|---|---|---|---|---|
| **1** | Beverage | <LOQ[1] | <LOQ | <LOQ | <LOQ | <LOQ | <LOQ |
| **2** | Beverage | <LOQ | <LOQ | <LOQ | <LOQ | <LOQ | <LOQ |
| **3** | Aloe beverage | <LOQ | <LOQ | <LOQ | <LOQ | <LOQ | <LOQ |
| **4** | Beverage | <LOQ | <LOQ | <LOQ | <LOQ | <LOQ | <LOQ |
| **5** | Aloe beverage | <LOQ | <LOQ | <LOQ | <LOQ | <LOQ | <LOQ |
| **6** | Beverage | <LOQ | <LOQ | <LOQ | <LOQ | <LOQ | <LOQ |
| **7** | Aloe beverage | **0.5** | **0.5** | **1.0** | <LOQ | <LOQ | <LOQ |
| **8** | Beverage | <LOQ | <LOQ | <LOQ | <LOQ | <LOQ | <LOQ |
| **9** | Aloe beverage | <LOQ | <LOQ | <LOQ | <LOQ | <LOQ | <LOQ |
| **10** | Aloe beverage | <LOQ | <LOQ | <LOQ | <LOQ | <LOQ | <LOQ |
| **11** | Aloe beverage | <LOQ | <LOQ | <LOQ | <LOQ | <LOQ | <LOQ |
| **12** | Aloe beverage | <LOQ | <LOQ | <LOQ | <LOQ | <LOQ | <LOQ |
| **13** | Beverage | <LOQ | <LOQ | <LOQ | <LOQ | <LOQ | <LOQ |
| **14** | Aloe beverage | <LOQ | <LOQ | <LOQ | <LOQ | <LOQ | <LOQ |
| **15** | Jam | <LOQ | <LOQ | <LOQ | <LOQ | <LOQ | <LOQ |
| **16** | Aloe beverage | <LOQ | <LOQ | <LOQ | <LOQ | <LOQ | <LOQ |
| **17** | Beverage | <LOQ | <LOQ | <LOQ | <LOQ | <LOQ | <LOQ |
| **18** | Solid food supplement | **29.5** | **29.9** | **59.4** | <LOQ | **19.3** | <LOQ |
| **19** | Liquid food supplement | <LOQ | <LOQ | <LOQ | <LOQ | <LOQ | <LOQ |
| **20** | Liquid food supplement | <LOQ | <LOQ | <LOQ | <LOQ | <LOQ | <LOQ |
| **21** | Liquid food supplement | <LOQ | <LOQ | <LOQ | <LOQ | <LOQ | <LOQ |
| **22** | Liquid food supplement | <LOQ | <LOQ | <LOQ | <LOQ | <LOQ | <LOQ |
| **23** | Liquid food supplement | <LOQ | <LOQ | <LOQ | <LOQ | <LOQ | <LOQ |
| **24** | Aloe beverage | <LOQ | <LOQ | <LOQ | <LOQ | <LOQ | <LOQ |
| **25** | Aloe beverage | <LOQ | <LOQ | <LOQ | <LOQ | <LOQ | <LOQ |
| **26** | Aloe beverage | <LOQ | <LOQ | <LOQ | <LOQ | <LOQ | <LOQ |
| **27** | Liquid food supplement | <LOQ | <LOQ | <LOQ | <LOQ | <LOQ | <LOQ |
| **28** | Solid food supplement | **742.3** | **385.9** | **1128.2** | **23.1** | **222.6** | <LOQ |
| **29** | Solid food supplement | **881.2** | **471.7** | **1352.9** | **27.5** | **259.7** | <LOQ |

| | | | | | | | |
|---|---|---|---|---|---|---|---|
| **30** | Solid food supplement | **28.2** | **23.2** | **51.4** | **0.9** | **8.2** | <LOQ |
| **31** | Solid food supplement | <LOQ | <LOQ | <LOQ | <LOQ | <LOQ | <LOQ |
| **32** | Solid food supplement | <LOQ | <LOQ | <LOQ | **213.4** | **43.1** | <LOQ |
| **33** | Solid food supplement | <LOQ | <LOQ | <LOQ | **0.8** | **36.4** | <LOQ |
| **34** | Solid food supplement | <LOQ | <LOQ | <LOQ | <LOQ | **1.0** | <LOQ |
| **35** | Solid food supplement | <LOQ | <LOQ | <LOQ | **3.0** | <LOQ | <LOQ |
| **36** | Solid food supplement | **66.4** | **35.4** | **101.8** | **5.9** | **51.4** | <LOQ |
| **37** | Solid food supplement | <LOQ | <LOQ | <LOQ | <LOQ | <LOQ | <LOQ |
| **38** | Herbal infusion | <LOQ | <LOQ | <LOQ | **23.5** | **104.4** | <LOQ |
| **39** | Herbal infusion | <LOQ | <LOQ | <LOQ | <LOQ | **2.0** | <LOQ |
| **40** | Herbal infusion | <LOQ | <LOQ | <LOQ | <LOQ | <LOQ | <LOQ |
| **41** | Herbal infusion | <LOQ | <LOQ | <LOQ | 12.5 | **4.1** | <LOQ |
| **42** | Herbal infusion | <LOQ | <LOQ | <LOQ | <LOQ | <LOQ | <LOQ |
| **43** | Herbal infusion | <LOQ | <LOQ | <LOQ | **24.2** | **6.3** | <LOQ |

[1]LOQ: 0.5 mg/kg
